# Functional characterization of Aquaporin-like genes in the human bed bug *Cimex lectularius*

**DOI:** 10.1038/s41598-017-03157-2

**Published:** 2017-06-12

**Authors:** Hitoshi Tsujimoto, Joyce M. Sakamoto, Jason L. Rasgon

**Affiliations:** 1 Department of Entomology, Center for Infectious Disease Dynamics and the Huck Institutes of the Life Sciences, Pennsylvania State University, University Park, PA 16832 USA; 2 Department of Entomology, Texas A& University, College Station, TX 77843 USA

## Abstract

The bed bug *Cimex lectularius* is a blood-feeding re-emerging annoyance pest insect that has the ability to transmit *Trypanosoma cruzi* under experimental laboratory conditions. Aquaporins (AQPs) are water channel proteins that are essential in biological organisms. *C*. *lectularius* are constantly exposed to water-related stress, suggesting that AQPs may offer novel control avenues. We identified and cloned four AQPs from *C*. *lectularius*, assessed tissue and lifestage-specific expression, and characterized biochemical functions *in vitro* and *in vivo*. We identified an efficient water-specific AQP (ClAQP1), two aquaglyceroporins (ClGlp1 and ClGlp2) and a homolog of *Drosophila melanogaster* big brain (ClBib). ClGlp1 was only functional when co-expressed with the water-specific AQP. Simultaneous RNAi gene silencing of ClAQP1 and ClGlp1 significantly reduced water and urea excretion post blood feeding. The Bib homologue was enriched in embryos, exclusively expressed in ovaries, and when silenced, dramatically increased bug fecundity. Our data demonstrate that AQPs have critical roles in excretion, water homeostasis and reproduction in *C*. *lectularius*, and could be potential targets for control in this notorious pest.

## Introduction

The human bed bug *Cimex lectularius* is an insect belonging to the order Hemiptera (true bugs) that requires vertebrate (usually human) blood for development, egg production and survival. The bed bug causes annoyance, allergic reaction associated with mild to severe dermatitis, and even mental health problems among humans^1–3^. Along with increasing concerns of re-emerging *C*. *lectularius* populations around the world, it has been recently demonstrated to be capable of laboratory transmission of the etiological agent for Chagas disease, *Trypanosoma cruzi* ^4^, although significant transmission potential in the field remains to be determined.

Bed bugs have been associated with humans for thousands of years, with extant records dating back to 400 BC^5, 6^. Despite near-eradication in the 1950’s, bed bug populations have been rapidly reemerging in industrialized countries during the last 15 years^3^. Reemerging bed bugs have been shown to be highly resistant to multiple classes of insecticides including pyrethroids, organochlorides, organophosphates, and carbamates. Multiple mechanisms for insecticide resistance have been detected from many field populations, ranging from knockdown resistance and modification of detoxification enzymes to up-regulation of cuticular proteins to reduce chemical penetration^3, 7^. Hence, novel strategies to control the bed bugs are urgently needed.

Bed bugs are known to survive 4 months to 2 years without feeding^8^. Bed bugs obtain almost all water from the blood meal^8^. Survival under desiccation conditions can be enhanced behaviorally by prolonged quiescence and aggregation to form clusters that promote group effects of water conservation. Water conservation is not the only water stress that bed bugs face. Bed bugs require a blood meal for each molt and for egg production, which can reach more than three times of the unfed body weight of an adult^9^ limiting movement and leading to increasing probability of being killed by the host or predators. Therefore, rapid excretion of unwanted water and solutes is also important for survival.

Aquaporin (AQP) water channels are transmembrane proteins that facilitate movement of water and selected solutes across biological membranes, and are found in all domains of organisms. There are two major AQP subclasses; water-selective AQPs, and aquaglyceroporins (Glps), which transport small uncharged solutes in addition to water molecules^10^. In insects, there is another subclass of AQPs described by structural similarity, Big brain (Bib) homologues. In *Drosophila melanogaster*, Bib does not transport water, but has a role in *Notch* signaling in neural development^11^ and is unique in having long N- and C-terminal tails in addition to six conserved AQP transmembrane domains^12^.

In this study, we identified and functionally characterized 4 AQP genes in *C*. *lectularius* (one classical AQP [ClAQP1], 2 Glps [ClGlp1 and ClGlp2] and one Bib [ClBib]). *In vitro* and *in vivo* analyses demonstrated a functional interaction between ClAQP1 and the Glp genes, particularly ClGlp1, whose function was obligately dependent on co-expression with ClAQP1. ClBib was exclusively expressed in the female ovary, and while its transport function could not be determined, *in vivo* experiments demonstrated a role for this gene in regulating bed bug reproduction. With the increasing importance of *C*. *lectularius* as potential Chagas vector^4^ as well as its importance as a household pest, results from this study will provide new insights into potential control strategies for this emergent pest.

## Results

### Identification of AQP-like genes

Recent full genome sequencing (not available at the time experiments were conducted) identified 6–8 AQP-like genes in the *C*. *lectularius* genome^13, 14^. From the transcriptome data available at the time^15, 16^, we identified four aquaporin (AQP)-like genes and obtained full-length cDNA sequences for all of them by RACE. GenBank accession numbers for the nucleotide sequences are shown in Table S1. We identified all three functional subclasses of AQPs (aquaporin [AQP], aquaglyceroporin [Glp] and big brain [Bib]) homologs in *C*. *lectularius*. Multiple alignment indicated highest similarity of AQP-like genes predicated in the VectorBase genome to the AQP-like genes we identified as follows: CLEC007784 (AQP2) with ClAQP1, CLEC025356 (AQP5) with ClGlp1, CLEC013397 (AQP4) with ClGlp2 and CLEC002337 (bib) with ClBib. None of them was identical at the amino acid level, indicating potential allelic differences or annotation error.

We identified a water-selective subclass of AQP in *C*. *lectularius* (ClAQP1). ClAQP1 shows highest amino acid sequence identity to *Drosophila melanogaster* CG7777 and *Anopheles gambiae* AgAQP1 water-selective AQPs. RACE identified three similar sequences for ClAQP1 that differed at the 3′ end. Mapping onto the genome, all mapped to scaffold 41 and were identified as splice variants, similar to *Anopheles gambiae* AgAQP1^17^. Two insect Glp-like sequences were identified in *C*. *lectularius* (ClGlp1 and ClGlp2). Both were most closely related to the recently postulated “Entomoglyceroporin” (“Eglp”) subclass evolved from water-selective AQPs such as AQP-Bom2 from the silk moth *Bombyx mori*, and PvAQP2 from the sleeping midge *Polypedilum vanderplanki* ^18, 19^. In the genome, ClGlp1 consists of 6 exons, and ClGlp2 consists of 5 exons. The Eglp subclass channels have been demonstrated to transport not only glycerol and urea, but also small polyols^19^. We identified a sequence similar to *D*. *melanogaster* big brain (Bib), which has putative functions in embryonic neural development^11^. This subclass of AQPs has long N-terminal and C-terminal cytoplasmic domains, and no reported water channel activity. ClBib consists of 7 exons.

### Phylogenetic analysis

**Figure 1:**
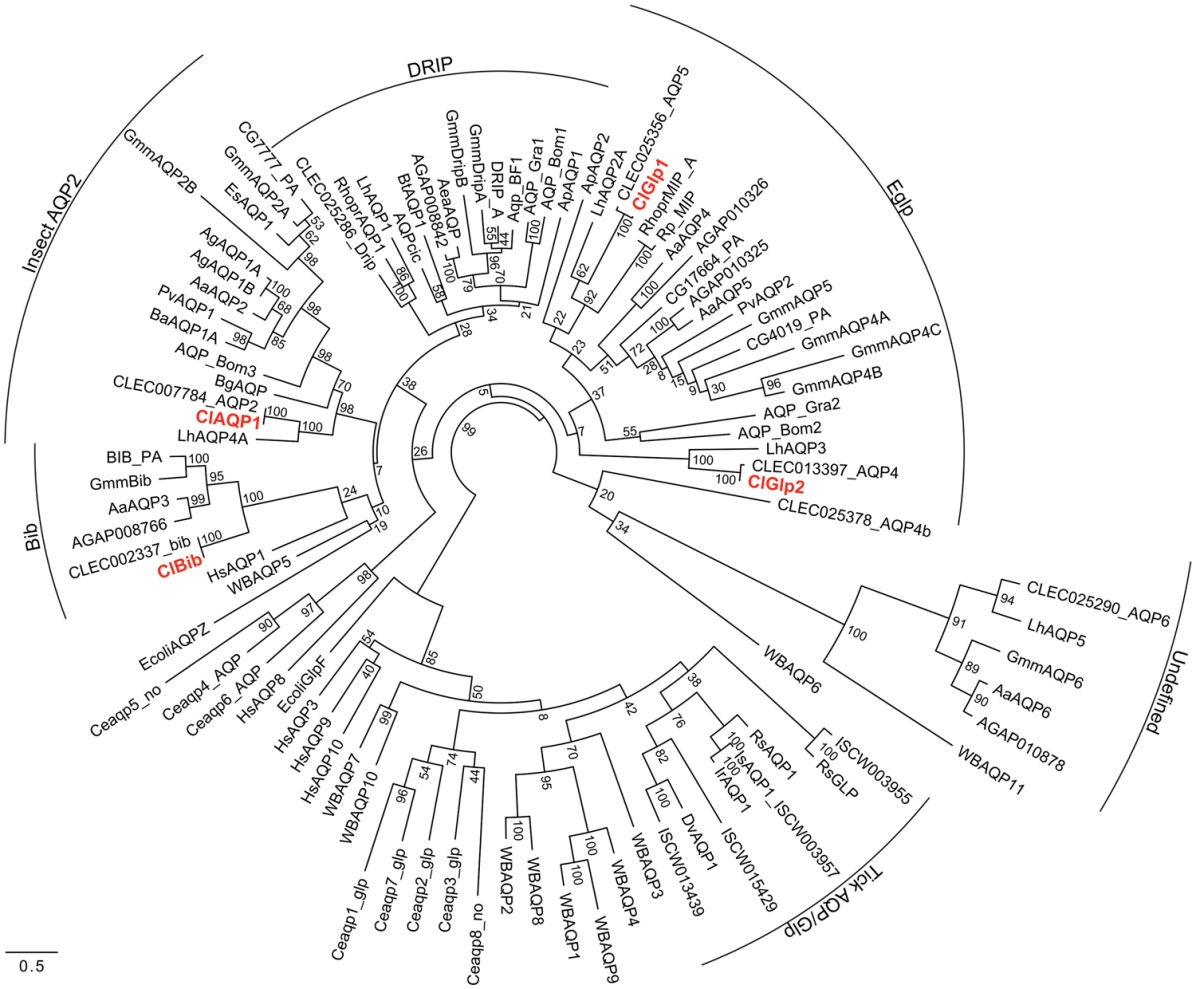
Maximum likelihood phylogenetic tree of identified *C*. *lectularius* AQP-like gene sequences (Red). Numbers at notes represent percent bootstrap values. Taxa accession numbers are listed in Table S1.

We examined relationships of these genes with other AQP genes using maximum likelihood phylogenetic analysis (Fig. 1). As expected, although bootstrap values were low, ClAQP1 clustered with *Drosophila* CG7777 and AgAQP1, and ClGlp1 and ClGlp2 clustered in the Eglp clade. Eglps cluster as sister clade to that containing *Drosophila* integral protein (DRIP) rather than traditional Glps as discussed by Finn *et al*.^19^. All the ClAQPs, except for ClBib, clustered as sister clade of other hemipteran AQPs.

### AQP expression

**Figure 2:**
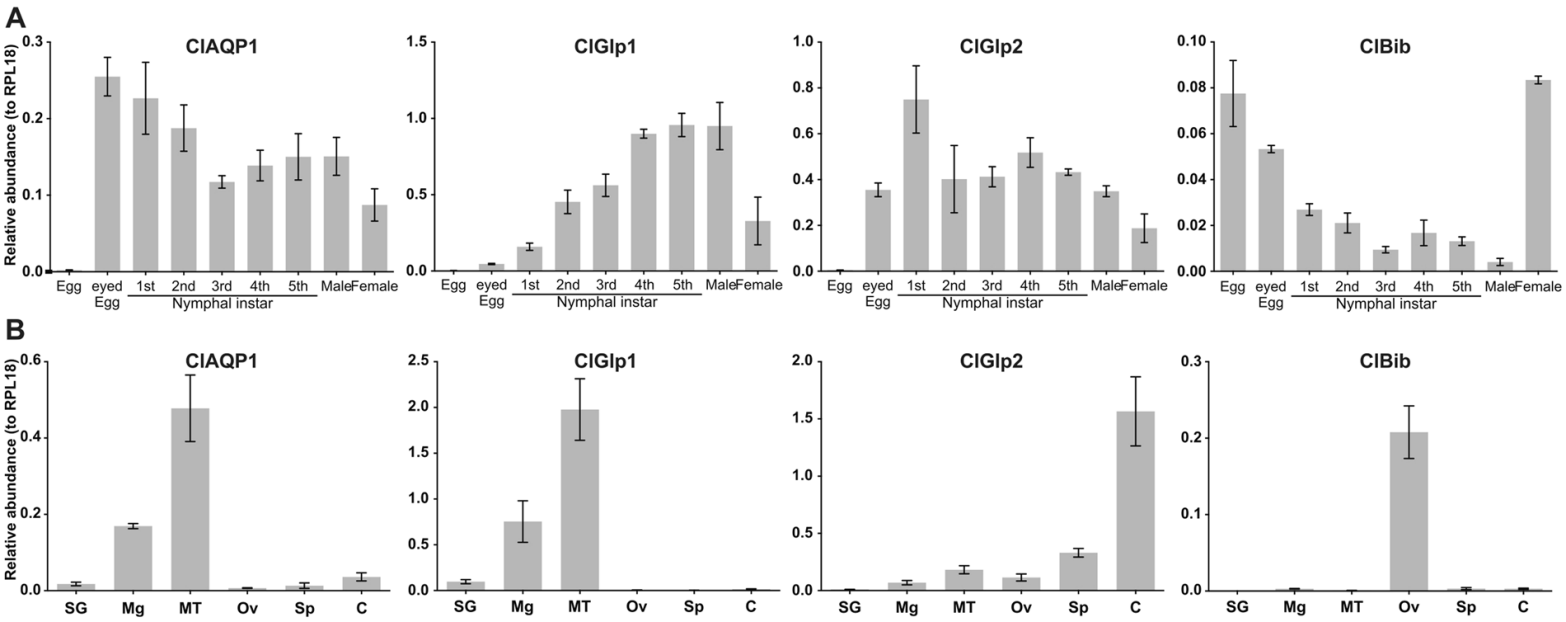
Lifestage and tissue-specific expression of *C*. *lectularius* AQP-like genes quantified by quantitative real-time PCR. (**A**) Lifestage-specific expression. (**B**) tissue-specific expression in adult female bugs. ClAQP1 and ClGlp1 have almost superimposable expression patterns, with primary expression in the gut and malpigian tubules. ClGlp2 is primarily expressed in the carcass. ClBib is only expressed in the ovaries. SG = salivary glands, Mg = midgut, MT = malpigian tubules, OV = ovaries, Sp = spermalege, C = carcass. The graph shows mean ± SD.

We determined expression patterns of ClAQP transcripts by quantitative real-time PCR (qRT-PCR). We first assessed expression in *C*. *lectularius* developmental stages (Fig. 2A). ClAQP1 is expressed throughout developmental stages except for the early embryo (“eggs”). ClGlp1 expression increases as developmental stages advance until the final instar nymph. ClGlp2 is expressed all stages except early embryo. ClBib is highly expressed in embryos and female adults. Next we examined tissue-specific expression in adult female organs (Fig. 2B). Remarkably, ClAQP1 and ClGlp1 exhibited almost superimposable tissue-specific expression and were abundantly expressed in the Malpighian tubules and midgut. ClGlp2 was expressed most abundantly in the carcass (which includes the epidermis, nervous system, fat body, hemocytes and tracheal system) suggesting possible functional divergence from ClGlp1. ClBib is expressed exclusively in the ovary.

### Channel activity in *Xenopus* oocytes

**Figure 3:**
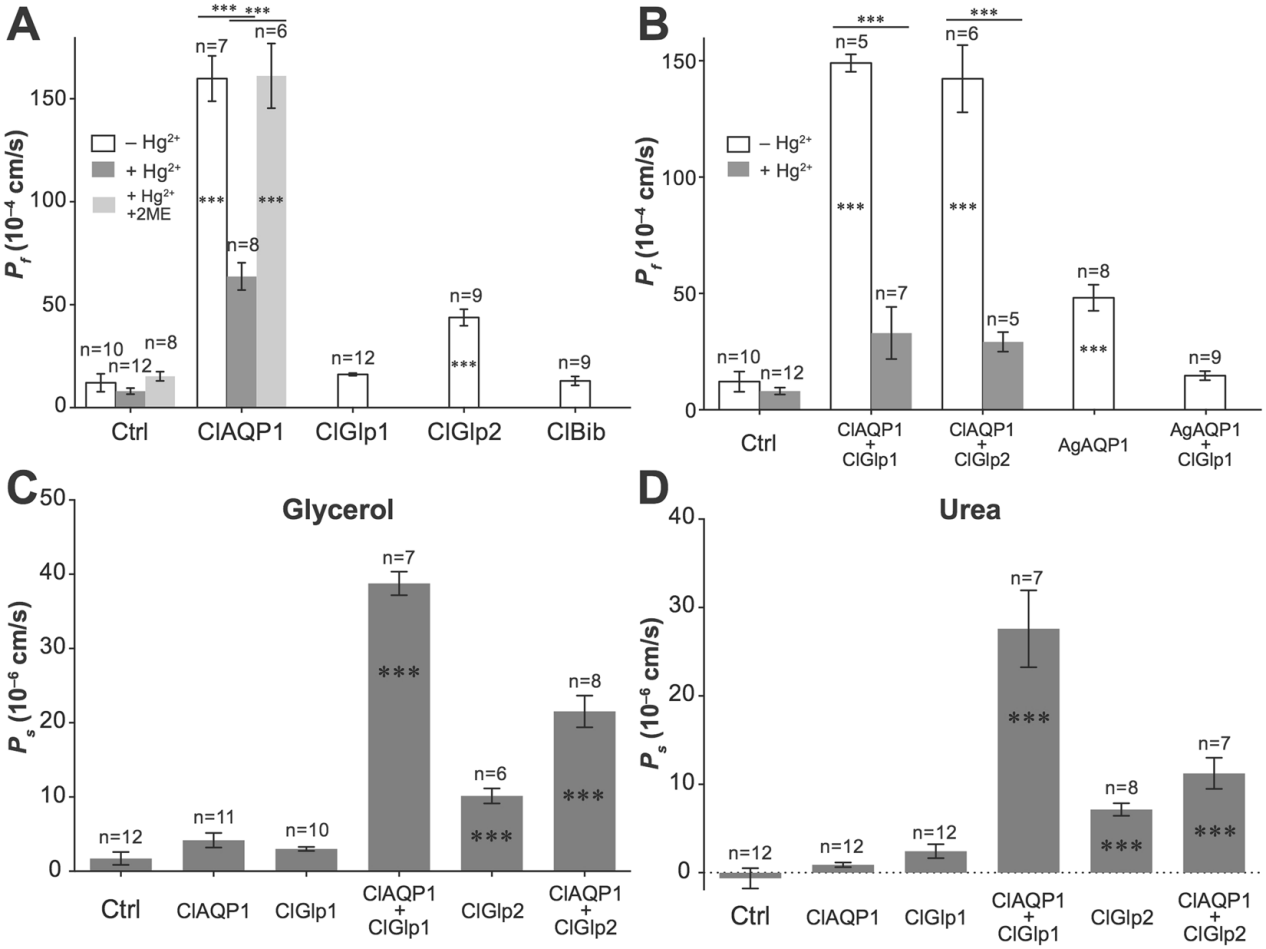
Water and solute transport activity of *C*. *lectularius* AQP genes expressed individually in *Xenopus* oocytes. (**A**) ClAQP1 transports water, and is inhibited by mercury. ClGlp1 and ClBib do not transport water. ClClp2 moderately transports water. (**B**) Water transport ability of ClAQP1 is not altered by co-expression with ClGlps. (**C**) ClGlp1 does not transport glycerol when expressed by itself, but showed high efficiency glycerol transporter activity when co-expressed with ClAQP1. Glycerol transport ability of ClGlp2 is enhanced when co-expressed with ClAQP1. (**D**) ClGlp1 does not transport urea when expressed by itself, but showed high efficiency urea transporter activity when co-expressed with ClAQP1. Urea transport ability of ClGlp2 is enhanced when co-expressed with ClAQP1. The graph shows mean ± SEM. Asterisks in the bars represent statistical significance (P < 0.001) in comparison to control; asterisks above the bars represent statistical significance (P < 0.001) comparing between the groups below the horizontal line.

We expressed putative AQP-like genes in *Xenopus laevis* oocytes and assessed water and solute permeability by swelling assay. High water permeability was observed only for ClAQP1, which was reversibly inhibited by Hg^2+^ (Fig. 3A). ClGlp2 exhibited significant, but low water permeability. We detected no significant water permeability for ClGlp1 or ClBib. We did not detect significant glycerol or urea permeability for any of the ClAQPs when expressed alone. However, due to the *in vivo* co-expression similarities of ClAQP1 and ClGlp1, we speculated that their function might depend on co-expression. When ClAQP1 was co-expressed with either ClGlp1 or ClGlp2, water permeability was unchanged and inhibited by Hg^2+^ (Fig. 3B). However, in contrast to ClGlp1 mono-expression, when ClGlp1 was co-expressed with ClAQP1 oocytes exhibited high glycerol and urea permeability (Fig. 3C and D). ClAQP1/ClGlp2 co-expression showed moderate increases in glycerol and urea permeability in comparison to ClGlp2 alone (Fig. 3C and D). In contrast, co-expression of ClGlp1 with AgAQP1 from *An*. *gambiae* (included as a control AQP from an unrelated insect) resulted in reduction of water permeability compared to AgAQP1 alone (Fig. 3B).

### RNAi knockdown of AQPs

**Figure 4:**
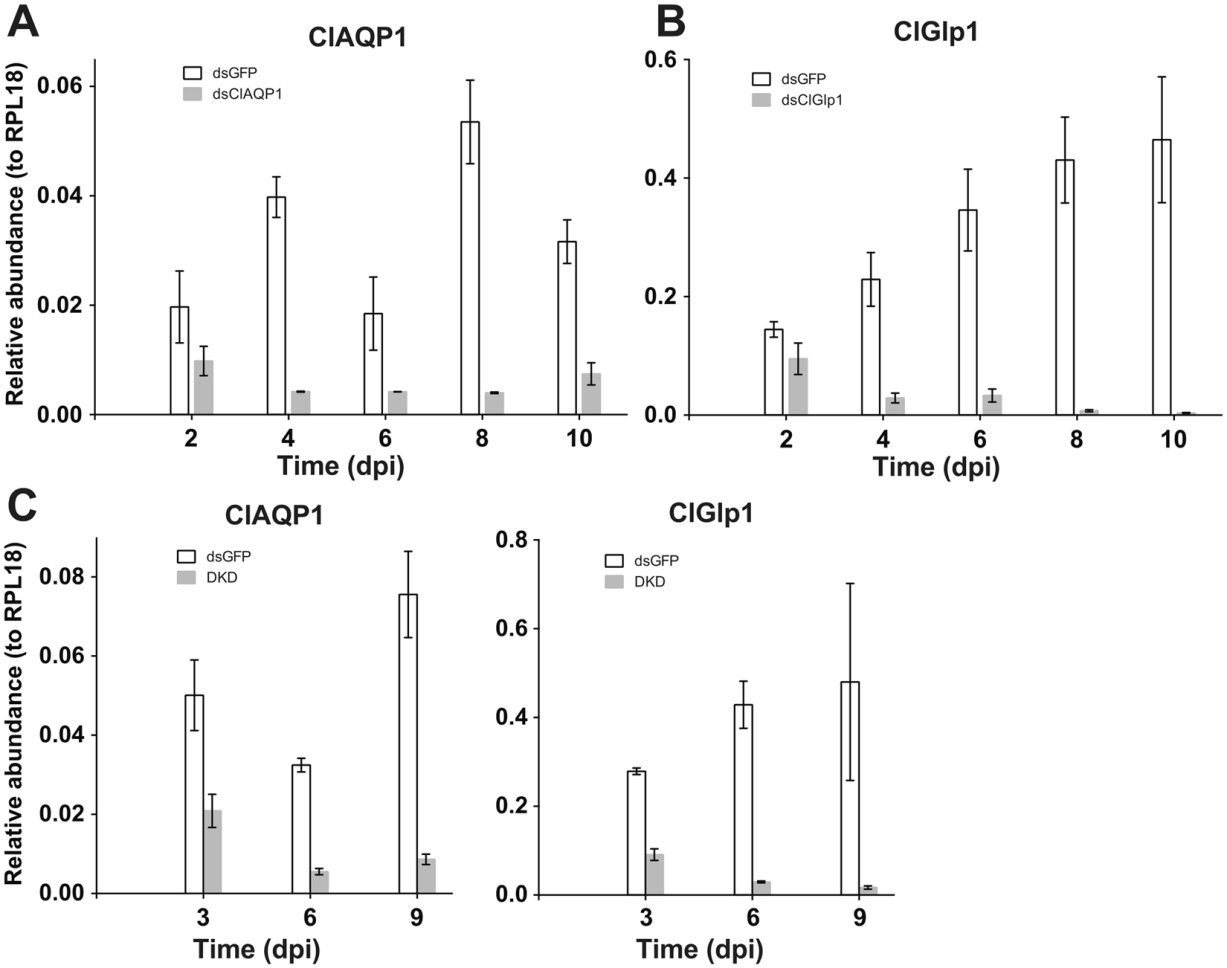
RNA interference (RNAi) is highly efficient in *C*. *lectularius*. Controls were injected with dsRNA against GFP. (**A**) RNAi of ClAQP1. (**B**) RNAi of ClGlp1. (**C**) Simultaneous knockdown of ClAQP1 and ClGlp1. DKD = double knockdown. The graph shows mean ± SD.

**Figure 5:**
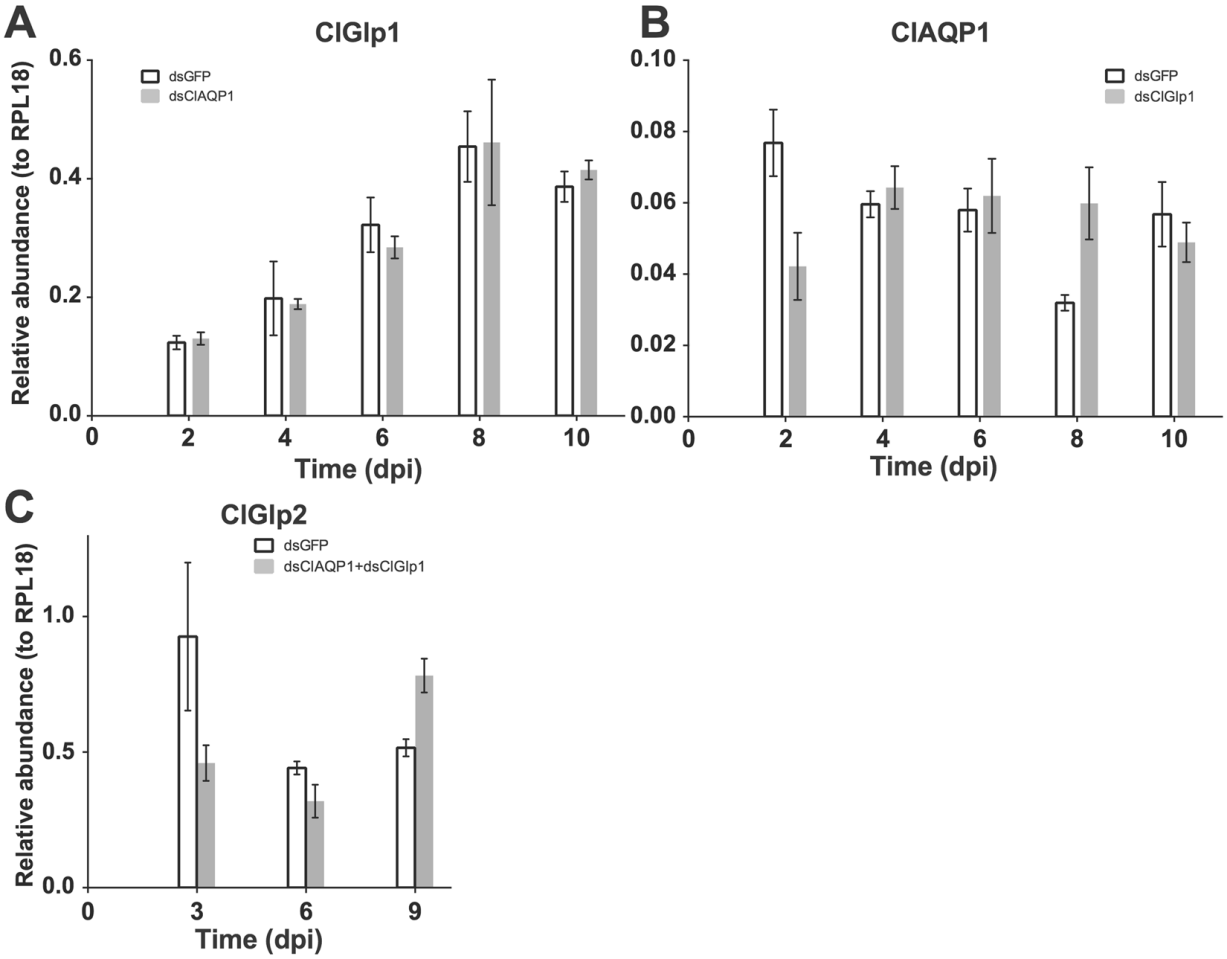
Lack of off-target effect of dsRNA injection. (**A**) ClGlp1 expression in dsClAQP1 (or control dsGFP) injected bed bugs. (**B**) ClAQP1 expression in dsClGlp1 (or dsGFP) injected bed bugs. (**C**) ClGlp2 expression in dsClAQP1/dsGlp1 (or dsGFP) injected bed bugs. Dpi: days post injection. The graph shows mean ± SD.

We first assessed RNAi efficacy in bed bugs. Significant reduction in mRNA transcript was observable beginning 4 days post-injection, and remained reduced 9–10 days post-injection (Fig. 4A,B). Double knockdown by injection of two (ClAQP1 and ClGlp1) dsRNAs also effectively reduced the transcripts of two genes (Fig. 4C). The reduction of the transcripts was specific as transcripts of non-target AQP genes were not affected (Fig. 5).

**Figure 6:**
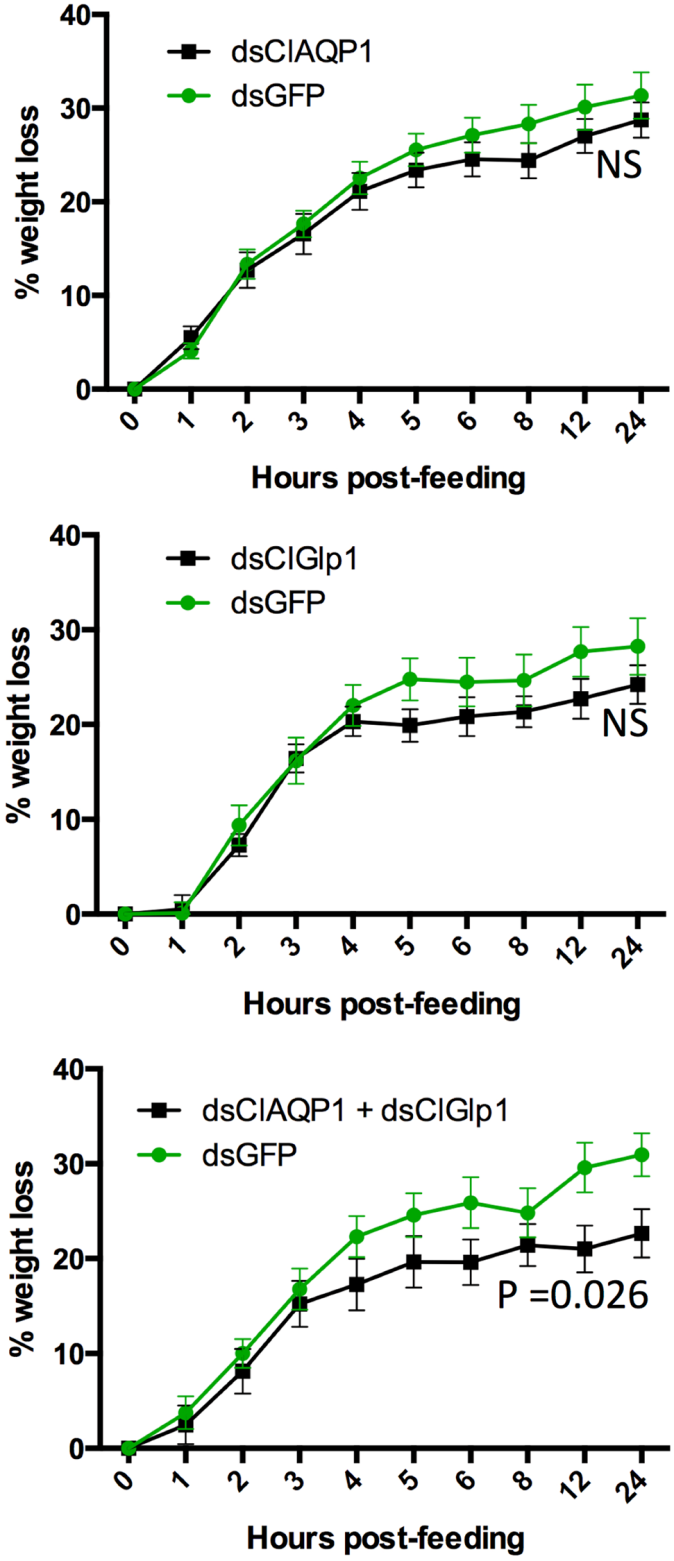
Effect of RNAi on bug excretion after a bloodmeal, as measured by weight loss. All controls were injected with dsRNA against GFP. (**A**) RNAi of ClAQP1. (**B**) RNAi of ClGlp1. (**C**) Double knockdown of ClAQP1 and ClGlp1. The graph shows mean ± SEM.

**Figure 7:**
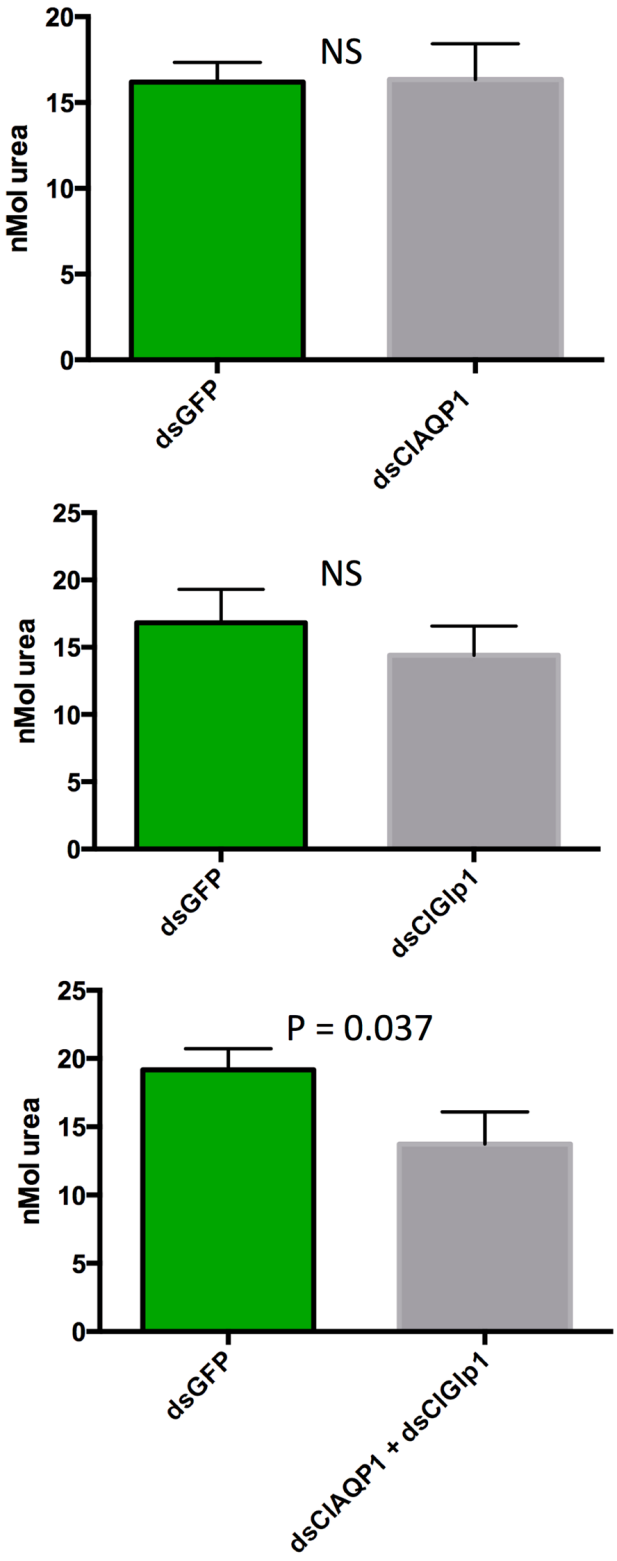
Effect of RNAi on urea excretion after a bloodmeal. All controls were injected with dsRNA against GFP. (**A**) RNAi of ClAQP1. (**B**) RNAi of ClGlp1. (**C**) Double knockdown of ClAQP1 and ClGlp1. The graph shows mean ± SEM.

We then tested the effects of RNAi on *in vivo* water excretion (measured by changes in weight) 24 hours after blood feeding. Single knockdown of ClAQP1 or ClGlp1 did not significantly reduce excretion compared to dsGFP-injected controls. However, upon simultaneous knockdown of both genes, excretion in treatment bugs was significantly impaired compared to controls, mirroring results from the swelling assay (Fig. 6). On the same bugs, we also quantified the excreted urea. Again, knockdown of ClAQP1 or ClGlp1 did not significantly affect urea excretion, but urea excretion was significantly reduced in the double knockdown treatment (Fig. 7).

### Potential role of ClBib in bed bug reproduction

**Figure 8:**
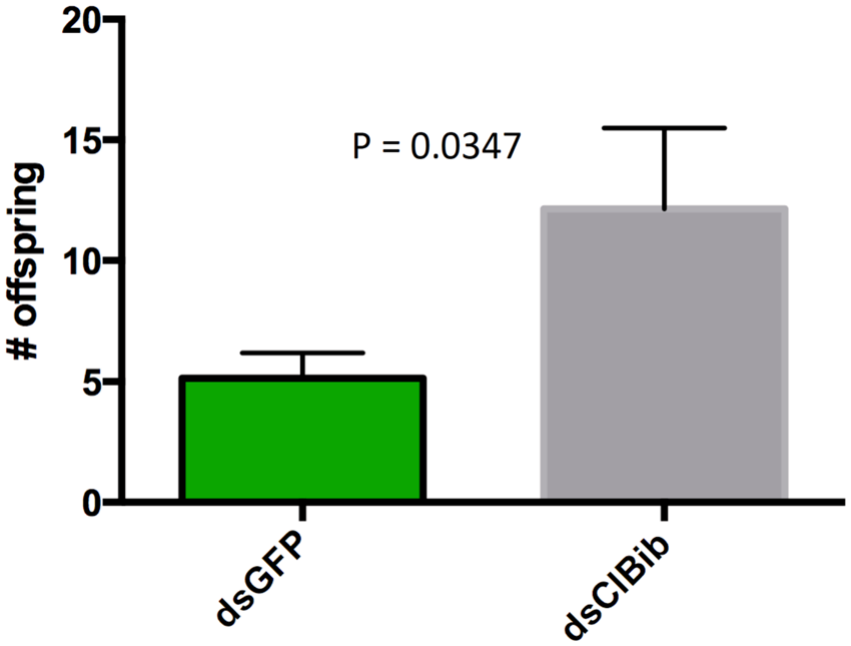
Effect of RNAi against ClBib on offspring production. Bugs with reduced ClBib expression oviposited significantly more eggs compared to GFP dsRNA-injected controls. The graph shows mean ± SEM.

Although we did not identify the transport capability (if any) of ClBib, the very tightly regulated expression of this gene in adult female ovaries suggested a potential role in reproduction. We knocked down expression of ClBib and assessed the effect of RNAi on egg production. Strikingly, bugs with reduced ClBib expression oviposited over twice as many eggs as GFP dsRNA-injected controls (Fig. 8), suggesting that ClBib acts as a negative regulator of reproduction in *C*. *lectularius*.

## Discussion

AQPs play important roles in insect homeostasis, such as diuresis and desiccation resistance in mosquitoes^20, 21^, dehydration, heat tolerance and intrauterine lactation in Tsetse flies^22^, and desiccation and cold tolerance in gall flies^23, 24^. We identified four AQPs in *C*. *lectularius* that included a water-specific AQP, two Glps and a Bib. Biochemical characterization revealed that ClAQP1 is a high efficiency water channel, while ClGlp2 acted as a low efficiency water channel (Fig. 3A). ClGlp1 did not detectably transport water, and did not transport glycerol or urea when expressed alone. However, when co-expressed with ClAQP1, it became a highly efficient channel for these solutes (Fig. 3C,D). Co-function of these 2 genes is further supported by their similar tissue-specific expression patterns (Fig. 2B) and results from *in vivo* functional assays (Figs 6 and 7). It should be noted that *in vivo* results might not be as clean as *in vitro* data due to the redundant effects of other AQP genes.

**Figure 9:**
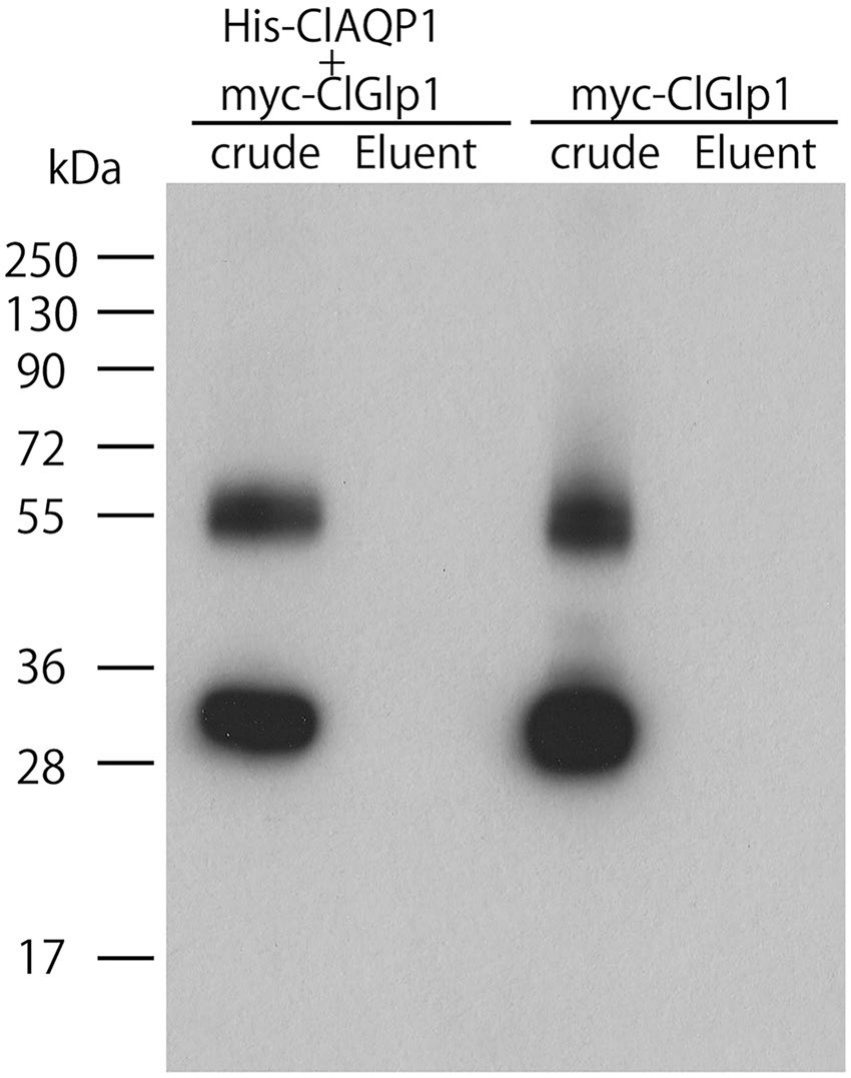
Pull-down assay to examine interaction between ClAQP1 and ClGlp1: *X*. *laevis* oocytes expressing His-ClAQP1 and myc-ClGlp1 or myc-ClGlp1 alone (control) were used to extract proteins, which were subjected to pull-down assay using Ni^2+^ NTA agarose matrix (Invitrogen). Presence of myc-ClGlp1 in the eluent was assessed by Western blot. Crude protein extracts were loaded as control.

Pull-down assays did not suggest a direct physical interaction between these 2 proteins (Fig. 9), suggesting that they act indirectly with one another, possibly by ClAQP1 driving an osmolality gradient that mediates solute transport via ClGlp1 (and to a lesser extent ClGlp2 (Fig. 3C,D). Further investigation into the detailed molecular interactions and transport mechanisms between these genes is warranted.

Knockdown of ClAQP1 and ClGlp1 significantly reduced bug water and urea excretion after a blood meal. These results suggest that ClAQP1 and ClGlp1 play critical roles in bug excretion. The bed bug faces challenges upon taking a blood meal, which can add up to three times or more of the unfed adults body weight^9^ For adult females we observed that blood meal increased the body weight by 318 ± 72% (mean ± SD) of pre-fed weight in average, similar to the observation by Araujo *et al*.^9^. This engorgement significantly limits the bugs’ mobility, which increases the danger of killing by the host or predation. Disruption of water and solute transport after blood feeding could lengthen the time of impaired mobility and may increase the chance of being caught by host or predators. It should be noted that phenotypic effects of gene silencing were not as extreme as the levels of transcript knockdown might suggest, possibly due to overlapping functions of uncharacterized AQPs.

Two AQPs have been characterized from the related insect, *Rhodnius prolixus*. One (RhoprAQP1) is similar to the DRIP subfamily and the other (Rp-MIP) to the Eglp subfamily^25–27^. Similar to our data, they are expressed in the Malpighian tubules, but their expression is not superimposable like ClAQP1 and ClGlp1^27^. Investigations into AQP function in related insects such as *R*. *prolixus* will be useful to compare AQP functional interactions in insects with similar dietary requirements.

Bed bugs are known to be able to survive many months without feeding, although their source of water is almost exclusively from blood^8^. In this context, control strategies that cause desiccation (boric acid, silica gel and diatomaceous earth) are demonstrated to be effective to control bed bugs, and desiccant dust formulation to treat bed bug infestation have been suggested as alternatives to conventional chemical insecticides^3^. Therefore, manipulation of AQPs could disrupt desiccation resistance, leading to novel control strategies.

Big brain homologues have not been conclusively demonstrated to transport water or other substrates, but may have a role in insect developmental processes^28^. We found based on both expression data and RNAi experiments that ClBib may have a role in regulating bed bug reproduction. In the adult female, ClBib is exclusively expressed in the ovary and is not detectable in other tissues (and in other stages, is most highly expressed in the early embryo) (Fig. 2). When ClBib expression was reduced by RNAi, the number of eggs oviposited dramatically increased (Fig. 8). This suggests that ClBib may act as a negative regulator of bed bug reproduction. There are well known tradeoffs between reproduction and survival^29^ and mechanisms to limit reproduction under some circumstances could be adaptive. Future studies investigating the effect of ClBib on egg hatch rate, and bug fitness using life table analysis are clearly warranted.

In this study, we identified all the subclasses of AQPs in *C*. *lectularius* and their functions. Our biochemical data for channel functions of these AQPs suggest that ClAQP1 and ClGlp1 functionally specialized as water channel and solute transporter, respectively, and that there is a functional interaction between these channels. Elevated expression of ClBib in embryo and ovary implies its role in embryogenesis. Knockdown of ClAQP1 and ClGlp1 resulted in significant reduction of excretion after blood feeding, which indicates that these genes play important roles in excretion and may be potential targets for controlling the bed bugs.

## Methods

All methods were carried out in accordance with Penn State University institutional guidelines and regulations.

### *Cimex lectularius* colony

The *Cimex lectularius* “field” strain (an amalgam of several populations collected from cities across the US in 2005^30^) was maintained at the Department of Entomology, Pennsylvania State University. Bugs were reared at 27 °C, 50% relative humidity and 14:10 (L:D) in plastic rearing jars with folded filter papers. Bugs were fed weekly on commercially purchased expired anonymous human blood using water-jacketed glass feeders in accordance with PSU IRB approval. Informed consent for anonymous blood donors was obtained by the blood bank.

### Identification of AQP genes

We obtained the contig set for *C*. *lectularius* transcriptome illumina reads described in ref. 15. We assembled another contig set from *C*. *lectularius* transcriptome 454 reads^16^ (SRR068315.1) using the MIRA assembler. We searched for AQP-like sequences by local blast with tblastx against the two contig sets as databases using AgAQP1A (accession No: JF342682.1) and RpMIP (accession No: AJ250342.1) as queries. Identified contigs were compiled into similar sequences by CAP3^31^. Based on these sequences, we designed RACE primers to obtain full-length mRNA sequences for each identified gene.

Whole bugs or dissected organs (for qRT-PCR experiments below) were homogenized in TRI reagent (Sigma Aldrich, St. Louis, MO) and total RNA extracted following the manufacturer’s suggested protocol. Extracted RNA was treated by DNase (TURBO DNAfree, Life Technologies, Carlsbad, CA) for 1 hr at 37 °C. First-strand cDNA synthesis was performed using 500–1000 ng of total RNA and oligo d(T)_20_ primer with Accuscript High Fidelity Reverse Transcriptase (Agilent Technologies, Santa Clara, CA) at 42 °C for 2 hrs.

Rapid Amplification of cDNA Ends (RACE) was performed essentially as described previously^17^. Briefly, RNA was extracted from all life stages (egg without eye spots [egg], egg with eye spots [eyed egg], 1st, 2nd, 3rd, 4th, 5th instars, adults of both sexes) using TRI reagent, and equal amounts of RNA from each stage was mixed for RACE. RNA processing for RACE was performed according to the manufacturer’s suggested protocol (GeneRacer, Life Technologies, Carlsbad, CA). RACE PCR products were separated by agarose gel electrophoresis, and bands excised for gel extraction using the QIAGEN Gel Extraction kit (QIAGEN, Valencia, CA). Gel-extracted PCR products were cloned into pJET1.2/blunt vector using the CloneJET PCR Cloning Kit (Fermentas, Glen Burnie, MD) and inserts sequenced to obtain the sequences.

### Mapping exons in genomic sequence

To determine the location and structure of identified AQP genes, we obtained the *C*. *lectularius* genome assembly from the i5K project web site (ftp://ftp.hgsc.bcm.edu/I5K-pilot/Bed_bug/genome_assemblies/Clec_Bbug02212013.genome.fa) and performed local blastn search using full-length ClAQP sequences as query, and manually adjusting locations of exons so that introns contained canonical splice signals (GT-AG).

### Phylogenetic analysis

We obtained AQP polypeptide sequences from GeneBank, FlyBase and VectorBase for alignment and phylogenetic analyses (accession numbers or gene IDs in Fig. 1 are listed in Table S1). Sequences were aligned using SeaView^32^ using Clustal Omega, and phylogenetic trees constructed using maximum likelihood implemented by PhyML 3.1 with 500 bootstrap replications.

### Expression analysis by quantitative real-time PCR (qRT-PCR)

For developmental stage analysis, eggs, nymphs (first, second, third, fourth and fifth instars), male and female adults were sampled for RNA extraction and cDNA synthesis. We collected eggs laid overnight as “eggs” and observed them daily until distinct red eye spots appeared, and were allowed to develop for two more days, (“eyed eggs”). Nymphs and adults were unfed for a minimum of two weeks. Sample sized used were 40–50 for eggs including eyed eggs, 30–45 for first instars, 25–30 for second instars, 10–15 for third instars, 10 for fourth instars, 5 for fifth instars and adults (for both male and female). Organ dissection was performed using 2 week old unfed adult females. Salivary glands, midgut, hindgut, Malpighian tubules, spermalege and ovaries were dissected from 50, 10, 10, 10 and 10 individuals, respectively in PBS and immediately pooled in TRI reagent. Five carcasses (whole body without salivary glands, gut system, Malpighian tubules, spermalege and ovaries) were also pooled. RNA extraction was performed immediately after dissection, otherwise samples were stored at −80 °C until used.

Primers were designed using the Primer3 server^33, 34^ to amplify 53–145 bp fragments of cDNA. All primer sets were empirically verified to have amplification efficiency (E) between 0.9 and 1.1 with the same qPCR protocol. cDNA was diluted 1/50 in nuclease-free H_2_O, of which 2.5 μL was used for 10 μL reactions using the RotorGene Q qPCR system (QIAGEN). Reactions were performed with 5 min at 95 °C, followed by 45 cycles of 95 °C for 5 sec and 60 °C for 10 sec and melt curve analysis from 50 °C to 95 °C. Expression was calculated relative to a housekeeping gene (ribosomal protein L18) by the −2^ΔΔCt^ method^35^. Primer sequences are listed in Table S2.

### Cloning, expression in *Xenopus* oocytes, and swelling assays

The aquaporin-like genes were cloned in the same manner as we previously described^16^. Coding sequences were amplified using primers containing restriction sites (EcoRI for ClAQP1, ClGlp1 and ClGlp2, or MfeI for ClBib for upstream primers and NheI for downstream primers) at the 5′ end. The clones used for full-length sequencing were used for PCR amplification by Ex Taq polymerase Hot Start Version (TaKaRa, Mountain View, CA). Agarose-gel purified PCR products were cloned into pJET1.2/blunt, and purified plasmids were digested by restriction enzymes. The digests were gel-purified and ligated into pXβG-myc. Purified constructs were linearized by XbaI for *in vitro* complementary RNA (cRNA) transcription with T3 RNA polymerase (Agilent Technologies, Inc., Santa Clara, CA). Synthesized cRNA was purified with the RNeasy Mini kit (Qiagen) and verified by agarose gel electrophoresis. *Xenopus laevis* ovaries were purchased from Xenopus 1 (Dexter, MI), defolliculated with collagenase I (Sigma) and injected with 5 ng of cRNA or 50 nL of nuclease-free water as control. After injection, oocytes were incubated for 3 days in ~200 mOsm Modified Barth’s Solution (MBS: 88 mM NaCl, 1.0 mM KCl, 2.4 mM NaHCO_3_, 15 mM Tris, 0.8 mM MgSO_4_, 0.4 mM CaCl_2_, 0.3 mM Ca(NO_3_)_2_, pH 7.6, containing 0.5 mM theophylline, 100 U/mL penicillin and 100 μg/mL streptomycin) for overexpression of aquaporins in the oocyte plasma membrane. Oocytes were subjected to swelling assay by transferring to MBS diluted to 70 mOsm. The change of oocyte volume was monitored at room temperature by a digital-camera equipped microscope for 60 seconds and the relative volume (*V*/*V*_*0*_) calculated from the cross-sectional area at the initial time (*A*_*0*_) and after a time interval (*A*_*t*_): *V*/*V*_*0*_ = (*A*_*t*_/*A*_*0*_)^3∕2^. The coefficient of osmotic water permeability (*P*_*f*_) was determined from the initial slope of the time course [*d*(*V*/*V*_*0*_)/*dt*], average initial oocyte volume (*V*_*0*_ = 9 × 10^−4^ cm^3^), average initial oocyte surface area (*S* = 0.045 cm^2^), the molar volume of water (*V*_*w*_ = 18 cm^3^/mol), and the osmotic solute gradient (osm_in_ − osm_out_: *P*_*f*_ = (*V*_*0*_ × *d*(*V*/*V*_*0*_)/*dt*)/(*S* × *V*_*w*_ × (osm_in_ − osm_out_)). A minimum of five individual oocytes were tested in each group.

To test the effect of Hg^2+^ for water permeability, oocytes were placed in 500 μM HgCl_2_ in MBS for 5 min prior to the swelling assay, and to test the reversibility of Hg^2+^-effect was assessed by transferring Hg^2+^-treated oocytes (500 μM, 5 min) to 5 mM 2-mercaptoethanol in MBS for 10 min.

Glycerol and urea permeability were assessed by replacing diluted MBS with MBS mixed with an equal volume of isosmotic (200 mOsm) glycerol or urea solution. The coefficient of solute permeability (*P*_*s*_) was calculated by the formula: *P*_*s*_ = (*V*_*0*_ × *d*(*V*/*V*_*0*_)/*dt*)/(*S* × *V*_*w*_ × (sol_in_ − sol_out_), where sol_in_ − sol_out_ is gradient of solute (glycerol or urea). Data were analyzed by T-tests for experiments with 2 treatments, otherwise were analyzed by one-way ANOVA with Tukey’s correction for multiple comparisons.

### Pull-down assay

To replace the myc tag with a 6-His tag for pull-downs, the ClAQP1-pXβG-myc plasmid was digested by BglII and NheI to remove the ClAQP1 insert with N-terminal myc tag. The insert was amplified using a forward primer containing the 6-His tag sequence in-frame with ClAQP1 coding sequence (CL456HisFBglIIa, Table S2) and the reverse primer used for ClAQP1 cloning into pXβG-myc. The digested insert was ligated into BglII/NheI-digested pXβG-myc (now lacking myc). Presence of the His tag was verified by Western blot using monoclonal anti-His antibody (abcam, Cambridge, MA) on membrane protein extracted from swelling assay-positive *X*. *laevis* oocytes.

*X*. *laevis* oocytes co-expressing ClAQP1-His and ClGlp1-myc were lysed in 0.1 × PBS with protease inhibitor cocktail (Roche, Indianapolis, IN) by repeated pipetting. After removing yolk components by centrifugation at 500 × g, 4 °C for 10 min, the membrane fraction was pelleted by centrifugation at 20,000 × g, 4 °C for 60 min. The membrane fraction pellet was dissolved in Pierce IP lysis buffer (Pierce, Rockford, IL) (1 mL for ~100 pooled oocytes) at room temperature overnight. Proteins were quantified using the BCA method (MicroBCA, Pierce) and ~250 μg of total protein mixed with 200 μL of Ni^2+^-NTA agarose matrix (Invitrogen, Carlsbad, CA) with addition of imidazole to a final concentration of 30 mM, and incubated at room temperature for 2 hrs. The protein- Ni^2+^-NTA agarose matrix mixture was applied on a column and washed 3 times with 30 mM imidazole containing 0.1% SDS, and elution performed by 5 aliquots of 200 μL of 200 mM imidazole, 0.1% SDS. Eluents were combined and concentrated using a 3000 MWCO Amicon Ultra filter (EMD Millipore, Billerica, MA) to ~50 μL, which was used for Western blot analysis using anti-myc-HRP antibody (Invitrogen) to probe myc-tagged ClGlp1.

### RNAi

For double-stranded RNA (dsRNA) synthesis, primers containing the T7 promoter sequence on the 5′ end were designed using e-RNAi^36^. dsRNA was synthesized with the MEGAscript T7 kit (Life Technologies) using purified PCR products as template at 37 °C overnight. Synthesized dsRNA was treated with RNase-free DNase (TURBO DNase, Life Technologies) at 37 °C for 15 min and purified using the MEGAclear Transcription Clean-Up kit (Life Technologies). Purified dsRNA was resuspended in 40 μL of nuclease-free water, concentration quantified by NanoDrop and stored at –80 °C in aliquots until use. dsRNA was injected in the same manner as previously described^37^. We used female bugs starved for at least two weeks for all RNAi experiments. 1 μg of dsRNA (approximately 210 nL) was injected into each bug using a Nanoject II microinjector (Drummond Scientific, Co., Broomall, PA). Knockdown efficiency on transcript levels was assessed by time-course qRT-PCR using 3 whole insects per time point per treatment. Control bugs were injected with dsRNA against GFP.

### Excretion assay

Blood feeding was performed at 7 days post dsRNA injection (dpi) using a water jacketed glass membrane feeder. Each bug injected with dsRNA was marked with paint and weighed individually before blood feeding. Bugs were allowed to feed for 10–20 min until repletion, immediately anaesthetized on ice, identified to treatment by paint marking, weighed, placed individually in a well of a 24-well plate, and weights monitored at 1, 2, 3, 4, 6, 8, 12, and 24 hr post blood meal. Change of weight ( = excretion) was expressed in percentage lost from gained meal weight by the equation: % excreted = ((W_tn_ − W_t0_)/W_gained_) × 100, where W_tn_ is weight at time n, W_t0_ is weight at time 0, and W_gained_ is the gained weight = the weight of the meal (W_t0_ − W_t–1_), where W_t–1_ is weight before blood meal. There was no significant difference in W_gained_ between any treatment (Fig. S1). Significant differences in weight 24 hours post-feeding between treatments were assessed by T tests.

### Urea quantification

After allowing bugs to excrete for 24 hrs, they were removed from the assay plate, each well filled with 100 μL of H_2_O and contents resuspended by pipetting. Solutions were transferred to centrifuge tubes and centrifuged at 10,000 × g at 4 °C for 5 min to remove erythrocyte-derived materials. Urea content was quantified using the Urea Colorimetric Assay Kit (Cat # K375-100, BioVision, Milpitas, CA) following the manufacturers suggested protocol using 20 μL of the excreta solution per 100 μL of final reaction volume. Significant differences in excreted urea between treatments were assessed by T-test.

### Reproduction assay

At 7 days post dsRNA injection (either dsClBib or dsGFP), mated female bugs were bloodfed using an artificial membrane feeder to repletion, and placed individually in a well of a 48-well plate. Five to six individuals were used for each treatment. The number of eggs laid per bug was monitored daily until oviposition ceased. Significant differences in the number of eggs produced between treatments were assessed by T-test.

## Electronic supplementary material

Supplementary Information
