# Supplementary material for: Functional characterization of Aquaporin-like genes in the human bed bug Cimex lectularius
Source: Sci Rep. 2017 Jun 12;7:3214. doi: 10.1038/s41598-017-03157-2 (PMC5468273; doi:10.1038/s41598-017-03157-2)
Supplement: Supplementary file 1 — Supplementary Information [file 41598_2017_3157_MOESM1_ESM.pdf]

Supplementary Information for: Functional characterization of Aquaporin-like genes in the human bed bug *Cimex lectularius*

Hitoshi Tsujimoto, Joyce M. Sakamoto and Jason L. Rasgon

Table S1: List of names and accession numbers/gene IDs for AQP and AQP-like sequences used for phylogenetic analysis.

**Name**	**Species**	**Acc#**	**Gene ID**
AgAQP1A	*Anopheles gambiae*	JF342682	
AgAQP1B	*Anopheles gambiae*	KR020501	
AgAQP2	*Anopheles gambiae*	KU055613	AGAP008842-PA
AgAQP3	*Anopheles gambiae*	AIT55906	AGAP010326-PA
AgBib	*Anopheles gambiae*	KU055612	AGAP008766-PA
AgGLP2	*Anopheles gambiae*	KU055614	AGAP010325-PA
AgAQP4	*Anopheles gambiae*	KU055615	AGAP010878-PA
AeaAQP	*Aedes aegypti*	AAF64037.1	
AaAQP2	*Aedes aegypti*	XP_001656932.1	
AaAQP3	*Aedes aegypti*	XP_001649747.1	
AaAQP4	*Aedes aegypti*	XP_001650168.1	
AaAQP5	*Aedes aegypti*	XP_001650169.1	
AaAQP6	*Aedes aegypti*	XP_001648319.1	
PvAQP1	*Polypedium vanderplanki*	BAF62090.1	
PvAQP2	*Polypedium vanderplanki*	BAF62091.1	
BaAQP1A	*Belgica antarctica*	BAK32935.1	
BgAQP	*Blattella germanica*	CBY77924.1	
DRIP-A	*Drosophila melanogaster*	ABA81817.1	
BIB-PA	*Drosophila melanogaster*	AAK93405.1	
CG17664-PA	*Drosophila melanogaster*	AAL39296.1	
CG7777-PA	*Drosophila melanogaster*	NP_725052.1	
CG4019-PA	*Drosophila melanogaster*	NP_611813.1	
GmmDripA	*Glossina morsitans morsitans*	AFP49892.1	
GmmDripB	*Glossina morsitans morsitans*	AFP49893.1	
GmmAQP2A	*Glossina morsitans morsitans*	AFP49894.1	
GmmAQP2B	*Glossina morsitans morsitans*	AFP49895.1	
GmmBib	*Glossina morsitans morsitans*	AFP49901.1	
GmmAQP4A	*Glossina morsitans morsitans*	AFP49896.1	
GmmAQP4B	*Glossina morsitans morsitans*	AFP49897.1	
GmmAQP4C	*Glossina morsitans morsitans*	AFP49898.1	
GmmAQP5	*Glossina morsitans morsitans*	AFP49899.1	
GmmAQP6	*Glossina morsitans morsitans*	AFP49900.1	
EsAQP1	*Eurosta solidaginis*	ACT34032.1	
Aqp-BF1	*Haematobia irritans exigua*	AAA96783.1	
AQP-Bom1	*Bombyx mori*	BAD69569.1	
AQP-Bom2	*Bombyx mori*	BAE97427.1	
AQP-Bom3	*Bombyx mori*	BAH29748.1	
AQP-Gra1	*Grapholita molesta*	BAH47554.1	

AQP-Gra2	*Grapholita molesta*	BAH47555.1	
AQPcic	*Cicadella viridis*	CAA65799.1	
ApAQP1	*Acyrthosiphon pisum*	ACL01373.1	
ApAQP2	*Acyrthosiphon pisum*	BAH71044.1	
BtAQP1	*Bemisia tabaci*	ABW96354.1	
Rp-MIP	*Rhodnius prolixus*	CAC13959.1	
RhoprMIP-A	*Rhodnius prolixus*	AEV57515.1	
RhoprAQP1	*Rhodnius prolixus*	AEV57513.1	
ClAQP1	*Cimex lectularius*	KT981949	
ClGlp1	*Cimex lectularius*	KT981953	
ClGlp2	*Cimex lectularius*	KT981954	
ClBib	*Cimex lectularius*	KT981952	
AQP6	*Cimex lectularius*		CLEC025290-PA
AQP2	*Cimex lectularius*		CLEC007784-PA
AQP5	*Cimex lectularius*		CLEC025356-PA
bib	*Cimex lectularius*		CLEC002337-PA
AQP4	*Cimex lectularius*		CLEC013397-PA
Drip	*Cimex lectularius*		CLEC025286-PA
AQP4b	*Cimex lectularius*		CLEC025378-PA
LhAQP1	*Lygus hesperus*	AHI85743.1	
LhAQP2A	*Lygus hesperus*	AHI85744.1	
LhAQP3	*Lygus hesperus*	AHI85749.1	
LhAQP4A	*Lygus hesperus*	AHI85750.1	
LhAQP5	*Lygus hesperus*	AHI85752.1	
RsAQP1	*Rhipicephalus sanguineus*	CAR66115.1	
RsGLP	*Rhipicephalus sanguineus*	CAX48963.1	
IrAQP1	*Ixodes rucinus*	CAX48964.1	
ISCW003957	*Ixodes scapularis*	KT988052	ISCW003957-PA
ISCW003955	*Ixodes scapularis*	XP_002399530	ISCW003955-PA
ISCW013439	*Ixodes scapularis*	XP_002400655	ISCW013439-PA
ISCW015429	*Ixodes scapularis*	XP_002399794	ISCW015429-PA
DvAQP1	*Dermacentor variabilis*	ABI53034.1	
Ceaqp1	*Caenorhabditis elegans*	NP_495510.1	
Ceaqp2	*Caenorhabditis elegans*	NP_495972.1	
Ceaqp3	*Caenorhabditis elegans*	NP_502044.1	
Ceaqp4	*Caenorhabditis elegans*	NP_505512.3	
Ceaqp5	*Caenorhabditis elegans*	NP_505691.2	
Ceaqp6	*Caenorhabditis elegans*	NP_001256246.1	
Ceaqp7	*Caenorhabditis elegans*	NP_508515.2	
Ceaqp8	*Caenorhabditis elegans*	NP_001024758.1	
WBAQP1	*Milnesium tardigradum*	AEP14555.1	
WBAQP2	*Milnesium tardigradum*	AEP14556.2	
WBAQP3	*Milnesium tardigradum*	AEP14557.1	

WBAQP4	*Milnesium tardigradum*	AEP14558.2
WBAQP5	*Milnesium tardigradum*	AEP14559.1
WBAQP6	*Milnesium tardigradum*	AEP14560.2
WBAQP7	*Milnesium tardigradum*	AEP14561.1
WBAQP8	*Milnesium tardigradum*	AEP14562.1
WBAQP9	*Milnesium tardigradum*	AEP14563.1
WBAQP10	*Milnesium tardigradum*	AEP14564.1
WBAQP11	*Milnesium tardigradum*	AEP14565.2
HsAQP1	*Homo sapiens*	NP_932766.1
HsAQP3	*Homo sapiens*	CAG46822.1
HsAQP8	*Homo sapiens*	AAH40630.1
HsAQP9	*Homo sapiens*	CAG46824.1
HsAQP10	*Homo sapiens*	NP_536354.2
EcoliAQPZ	*Echerichia coli*	NP_415396.1
EcoliGlpF	*Echerichia coli*	NP_418362.1

Table S2: Custom-made primers used in this study. Primers were named by original contig names: 456, ClAQP1; 2415, ClGlp1; 126, ClGlp2; 5911, ClBib.

Name	Sequence 5′-3′	Note
GSPCL456R1	CCCGAGGATGGTTTTCAGAGTGCTTTCTTT	5′RACE
GSPCL456R2	TGGTTTTCAGAGTGCTTTCTTTATCCGGCA	
GSPCL456R3	CAGAGTGCTTTCTTTATCCGGCATTTTTTTAGTA	
GSPCL2415R1	CGATCTTTATGTTGTTGTCCGCAGCACACT	
GSPCL2415R2	TTGTTGTCCGCAGCACACTCGACCGA	
GSPCL2415R3	AGCACACTCGACCGACAGCTTCGCAT	
GSPCL126R1	AAAGCAGTGCCCAACATCTCAGCGA	
GSPCL126R2	TGCCCAACATCTCAGCGAGTGCGAA	
GSPCL126R3	ATCTCAGCGAGTGCGAACCCGATCA	
GSPCL5911R1	AGGTGGCAGGAGCCTCGGCGTGAT	
GSPCL5911R2	GCAGGAGCCTCGGCGTGATAGGCG	
GSPCL5911R3	CTCGGCGTGATAGGCGCCTCTCGT	
GSPCL456F1	CGCGGCTGTCCTGTACGCCACACTT	3′RACE
GSPCL456F2	GTCCTGTACGCCACACTTTTCGCTGCTT	
GSPCL456F3	CCACACTTTTCGCTGCTTCACCACCCA	
GSPCL2415F1	TTCGGGGTCGATCAAGTTCGGTACTGT	
GSPCL2415F2	CGATCAAGTTCGGTACTGTCATCATCGCGA	
GSPCL2415F3	GTTCGGTACTGTCATCATCGCGATGTCCTT	
GSPCL126F1	CCCATCAGGTTGCAAAAGGAAGCACACC	
GSPCL126F2	GGTTGCAAAAGGAAGCACACCATTTTCAA	
GSPCL5911F1	AACCCCCAGCAGTACAGGGAATCACCA	
GSPCL5911F2	CCAGCAGTACAGGGAATCACCAAATCCACA	
GSPCL5911F3	TCACCAAATCCACAGTTCTCTAGGACGGA	
CL456FLUA	TCGTGGGGCATCGATCAAGTACAAACA	Full-length sequencing
CL456FLDA	AGAATTGTGGTTCAGAAATTGGACAAAAGCAA	
CL2415FLUA	TGTCGCCTATTCAACTAAATCAGTCACGTTC	
CL2415FLDA	AAAAAGGTGGTTTAATTTTCCTTGTTGAAGCAGT	
CL126FLUA	ACACGGACAGGACTCAGGAGAAGG	
CL126FLDA	TGTGCTTCCTTTTGCAACCTGATGGG	
CL5911FLUA	GCTTCGTCCGGTTCGGTATGGCTT	
CL5911FLDA	AGGCATCATTAATAAATGTCCTTCTTTGCCAGT	
CL456FEcoRIa	ataGAATTCcATGCCGGATAAAGAAAGCA	Cloning into pXβG-myc vector
CL456RNheIa	ataGCTAGCTCAGGCGTTCCCGTCGTT	
CL2415FEcoRI	ataGAATTCcATGGCCTATTTGCAAGTCT	
CL2415RNheI	ataGCTAGCTTAATTTTCCTTGTTGAAGCA	
CL126FEcoRI	ataGAATTCcATGCCCAGAAGGAAAGCAA	
CL126RNheI	ataGCTAGCTCAGATCGAATGGACCTCCT	
CL5911FMfeI	ataCAATTGgATGGCTTCGGGAGCTCTGA	
CL5911RNheI	ataGCTAGCTCAATATTGCTGATTTGGTGATTCCT	
CL456HisFBglIIa	ataAGATCTGATATCATGCACCATCATCATCATCATGAATTCATGCCGGATAAAGAAAGCA	
qCLRPL18F	GGAAGAGGAATGCTCGGGAGGCTGT	qRT-PCR
qCLRPL18R	GCTTCGTGTGCGAGCGAGGGG	
qCL456F	TGGCACAACAGCTTTGGGTAAGGACA	
qCL456R	TCAAGACGAGGAGGAAACCGAGCA	
qCL2415F2	GTGGTCGGCGTCGGCCTTG	
qCL2415R2	GGACCGAAGAGCTCAGGTGGTG	
qCL126F	AGGACGACATGCCCAGAAGGA	
qCL126R	CCCAGGCCGACGAAGAAAGCA	
qCL5911F	CCCACGACCTGGACCCCTTG	
qCL5911R	TGCACCGAGCCTCTGTTGGTT	
dsCL456F2	taatacgactcactatagggAATGCCGGATAAAGAAAGCA	dsRNA template
dsCL456R2	taatacgactcactatagggTGTTGTGCCAAGAGTTGCTC	
dsCL2415F	taatacgactcactatagggGGAGGGAATTCGGTTCTAGC	
dsCL2415R	taatacgactcactatagggGTAGACGAAGACCATCGGGA	

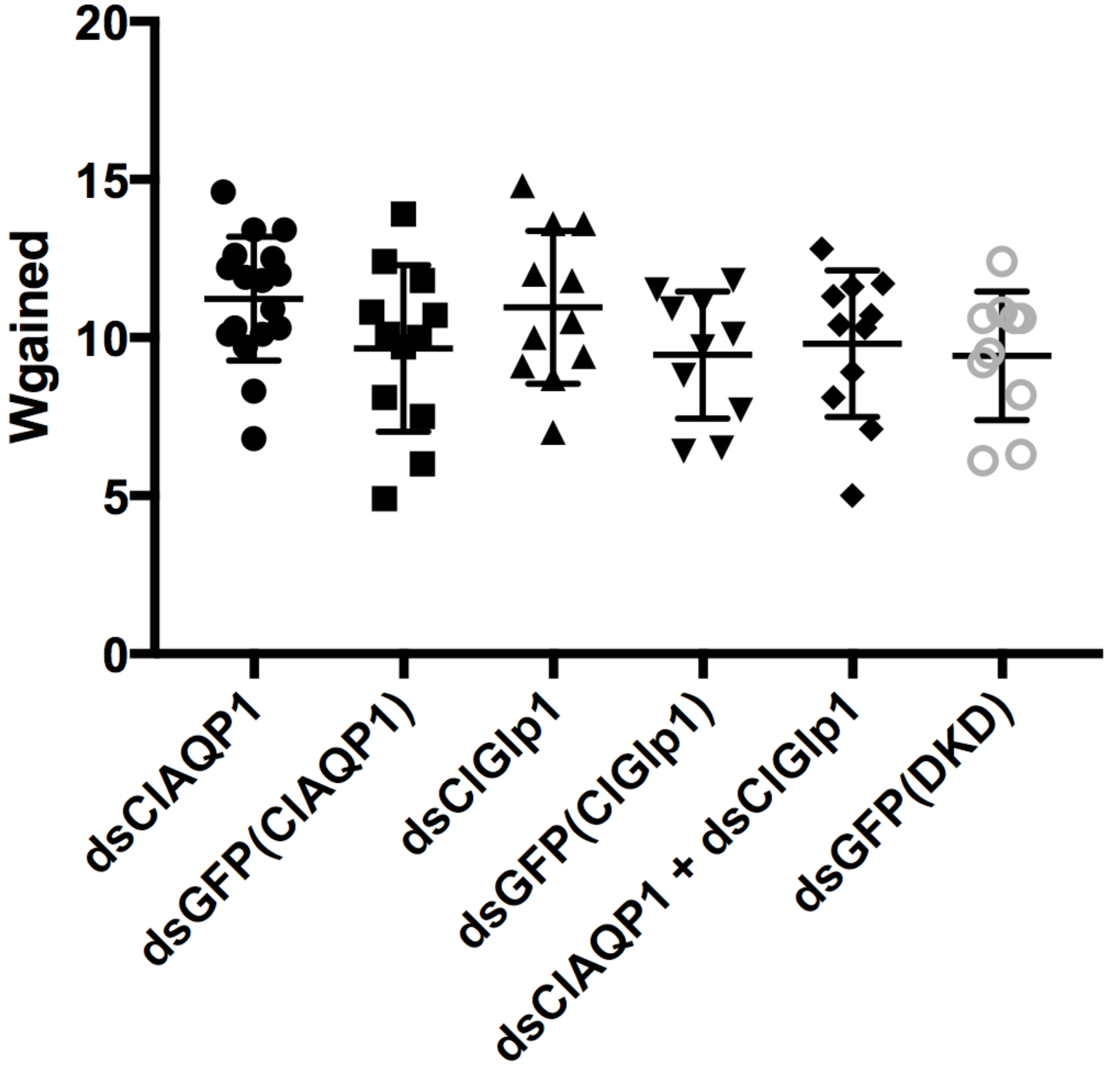

Figure S1. W_{gained} between treatments in the excretion assay. There was no significant difference in W_{gained} (i.e. blood meal size) between treatments (ANOVA).
